# Cardiovascular magnetic resonance is feasible in many patients aged 3 to 8 years without general anesthesia or sedation

**DOI:** 10.1186/1532-429X-16-S1-P377

**Published:** 2014-01-16

**Authors:** Ahmed E Kharabish, Naira Mkrtchyan, Christian Meierhofer, Stefan Martinoff, Peter Ewert, Heiko Stern, Sohrab Fratz

**Affiliations:** 1 Pediatric Cardiology and Congenital Heart Defects, German Heart Center, Munich, Germany; 2 Radiology and Nuclear Medicine, German Heart Center, Munich, Germany

## Background

Cardiovascular Magnetic Resonance (CMR) of pediatric patients has become routine clinical practice. Patients under eight years are usually examined under general anesthesia (GA) or sedation without intubation. However, in our clinical experience, CMR may be feasible in patients aged 3 to 8 years without general anesthesia or sedation without intubation.

## Methods

Retrospectively studied datasets of total number of 71 patients aged between 3 and 8 years. The total cohort was divided into two groups, first with no general anesthesia or sedation without intubation (no GA or sedation) and the second group patients with general anesthesia or sedation without intubation (GA or sedation). The patients' age groups and scan durations for each group, percentage of successfully answering the clinical question in each group, total number of scanned sequences in each group, and number of sequences per study were recorded and compared between both groups.

## Results

Forty-four patients in the no GA or sedation group, 27 in the GA or sedation group. The scan duration was in the no GA or sedation group: 35 minutes ± 20.44 minutes, and in the GA or sedation group: 60 minutes ± 31 minutes (p < 0.001). The percentage of successful reports was 95% (42 of 44) in the no GA or sedation group and 89% (24 of 27) in the patients with GA or sedation group.

## Conclusions

In scope of understanding the CHD hemodynamics, and clinical requests and questions; the decision of using neither general anesthesia with intubation nor sedation with without intubation was favored by our center's CMR unit over examining the patients under general anesthesia or sedation, to avoid the effect of general anesthesia and sedation on cardiac function during CMR, decrease the CMR scan duration, and increase the feasibility of the CMR in the young age group between three and eight years.

## Funding

All authors have no conflict of interest.

